# Malignancy with unknown primary presenting as acute cardiac tamponade: a case report

**DOI:** 10.4076/1757-1626-2-8176

**Published:** 2009-06-18

**Authors:** Edward J Banham-Hall, Awais M Bokhari

**Affiliations:** 1 Department of Acute Medicine, Ipswich Hospital NHS TrustHeath Road, Ipswich IP4 5PDUK; 2 Department of Cardiology, Bedford Hospital NHS TrustKempston Road, Bedford, MK42 9DJUK

## Abstract

A case report of a patient presenting in cardiac tamponade that was subsequently diagnosed as being secondary to malignancy of unknown primary. The patient was treated by urgent pericardiocentesis, followed by subsequent formation of a subxiphoid pericardial window. He was discharged home and given palliative chemotherapy. Malignant pericardial effusions are common, but it is rare for a patient to present in cardiac tamponade as the presenting feature of an unidentified malignancy. The causes, diagnosis and treatment of cardiac tamponade are discussed.

## Case presentation

A 65 year old Caucasian man presented to A&E with a five day history of progressively worsening diffuse chest pain radiating to the back. The pain was described as a dull ache, and had been associated with shortness of breath and a cough productive of white frothy sputum. There was an additional history of orthopnoea, and on direct questioning he did admit to some recent bipedal oedema.

His past medical history comprised only of epilepsy, and the only cardiac risk factor was a 40 pack-year smoking history. His only medication was phenytoin 100 mg BD. There were no drug allergies or pertinent family history. Review of systems was unremarkable.

**Figure 1.**
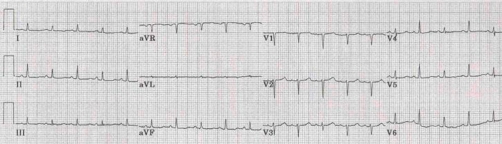
ECG on admission. The admission ECG demonstrating slightly reduced QRS complexes.

Clinical examination revealed a JVP raised 6 cm, a scattered wheeze audible throughout the precordium and quiet heart sounds but was otherwise unremarkable. ECG showed a sinus tachycardia at 120 bpm and low voltage complexes (Figure 1) Chest x-ray demonstrated a large globular heart.

**Figure 2.**
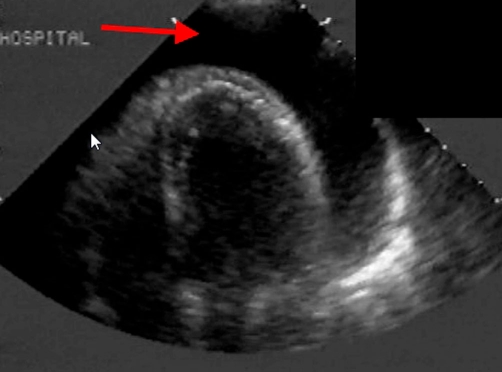
Echocardiographic image. Confirmation of a large pericardial effusion shown on this echo image by the red arrow.

The clinical impression formed was of a pericardial effusion, and an echocardiogram performed urgently confirmed the presence of a 4 cm global pericardial effusion with evidence of tamponade (Figure 2).

**Figure 3.**
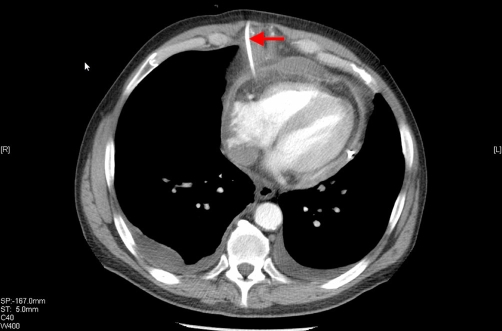
CT scan. Successful siting of the pericardial drain was seen on CT with consequent resolution of the pericardial effusion.

An urgent pericardiocentesis was performed and a drain was inserted under fluoroscopic guidance. 300 ml of blood-stained fluid was aspirated, and imaging confirmed a reduction in the effusion size to less than 20 mm (Figure 3). The fluid was sent for biochemistry, microbiological analysis, and cytology.

Over the following 36 hours a further 800 ml of pericardial effusion was removed via the drain before it was removed.

Fluid sent for cytology contained numerous lymphocytes together with malignant cells consistent with material from an adenocarcinoma or mesothelioma. Subsequent immunocytochemical analysis was performed and the tissue was found positive to pCEA, EMA, BerEP4, Calretinin and CK7. It was negative to Desmin, CK20, and TTF-1. These results were reported as consistent with a lung primary but also raised the potential of an extra-pulmonary primary.

**Figure 4.**
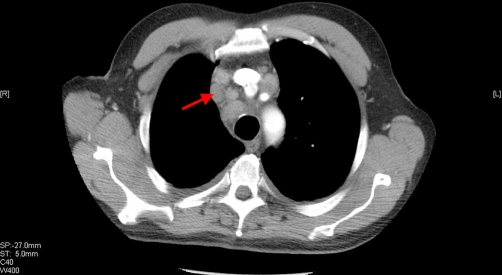
CT scan. Perihilar lymphadenopathy demonstrated on CT in keeping with metastatic carcinoma.

A staging CT showed extensive mediastinal lymphadenopathy with spread to the pre-aortic, para-tracheal and subcarinal nodes (Figure 4). There was an irregular lobulated mass in the right upper lobe measuring 2.3 cm at its widest diameter. The pericardium was thickened, suggestive of metastatic involvement. There was no evidence of disease below the diaphragm. The radiological differential diagnosis was of metastatic bronchial carcinoma, or of a disseminated unknown primary with a pulmonary metastasis.

In view of the malignant cells found in the fluid cytology the patient was referred for a subxiphoid pericardial window. This was performed without complication and samples of pericardium were sent for histology.

Histological analysis of the pericardial tissue demonstrated fibrosis and mild inflammation, without infiltration by malignant cells. The report indicated that these findings were non-specific.

The patient and results were discussed at the regional lung cancer MDT at which the decision was taken to treat with palliative chemotherapy for an unknown primary.

## Discussion

Malignant pericardial effusions are well recognised in malignancy [1,2], and need to be diagnosed early in light of their life-threatening nature should tamponade occur. Various cases have been reported with many primary malignancies and the best course of oncological management is a source of some debate [3,4]. It is however much more uncommon for the pericardial effusion to be the presenting feature of a hitherto undiagnosed malignancy with literature searches revealing very few reported such presentations [5].

Malignant pericardial effusion sufficient to require drainage is a poor prognostic factor, with reported median survival of 6.1 months [4].

Other common causes of pericardial effusions include acute myocardial infarction, PCI, uraemia, tuberculosis, infection, connective tissue disorders and trauma. The aetiology is unknown in 40 - 85% of cases [6].

Patient's presentation and development of symptoms depends on three principle factors - the volume of the pericardial effusion, the rate of accumulation and the elasticity of the pericardium. Larger volume effusions are better tolerated if the rate of accumulation is slow and the pericardial elasticity is high.

Patients do infrequently present in a state of haemodynamic embarrassment secondary to pericardial tamponade from their effusion. Clinical parameters suggesting this include hypotension, tachycardia, elevated JVP, quiet heart sounds, oliguria, and pulsus paradoxus. A friction rub is pathognomonic but frequently absent. ECG changes include low voltage complexes and ST segment changes that can mimic those seen in pericarditis. The diagnosis is confirmed with echocardiography, and findings indicating tamponade include diastolic collapse of the right atrium or ventricle with respiratory Doppler variation in transvalvar flows [7].

The treatment of cardiac tamponade is drainage of the effusion. Medical measures should only be utilised whilst arrangements for this are made and should not be viewed as alternatives. Intravenous resuscitation in the volume deplete patient may boost right heart filling pressures, whilst mechanical ventilation increases intrathoracic pressure thus impeding right sided filling pressures and can therefore be counter-productive. As adrenergic activation is already high in tamponade inotropic agents are not regarded as beneficial but are frequently trialled [8,9].

Pericardiocentesis should be carried out in a cardiac catheter laboratory by experienced staff with appropriate nursing and technical support. Rarely clinical urgency will necessitate “blind” intervention in less than ideal facilities. This should be regarded as only being indicated in an absolute emergency. A 15 cm 18 gauge pericardiocentesis needle should be inserted just left of the xiphoid process until just behind the bony ribcage. It should then be angled at about 20^o^ to the abdominal wall, aiming for the left shoulder tip. Lignocaine should be infiltrated as the needle is advanced, and repeated aspirations should be rewarded with a feeling of “give” once access to the pericardial sac is achieved. Complications include pneumothorax, arrythmias, ventricular laceration, and pyopericardium [10].

Pericardial window formation prevents pericardial fluid from reaccumulating following the removal of a pericardiocentesis drain. This can be done by thoracoscopy or by a subxiphoid approach. Where thoracoscopy would be preferred but is unavailable as minithoracotomy may be used instead. The thoracoscopic technique permits visualisation of the pericardium and pleura to allow adequate tissue to be sampled for histology when the underlying cause of the effusion is uncertain, and is generally preferred for when pleural drainage is needed as well. However, in malignant pericardial effusions it risks contamination of the pleural space with cancerous cells and a subxiphoid approach is utilised. A retrospective analysis comparing subxiphoid and thoracoscopic techniques found they had similar recurrence rates, postoperative complications, lengths of stay and need of intensive care unit admission [11].

**Teaching Points:**
Pericardial effusions are recognised complications of malignancy;
Initial presentation of a malignancy with a pericardial effusion is unusual;
Presentation with cardiac tamponade is a medical emergency requiring prompt and expert treatment;
Pericardiocentesis is a safe procedure in expert hands with echocardiographic or fluoroscopic monitoring;
Blind Pericardiocentesis should only ever be attempted in an absolute emergency.

## List of abbreviations

None.
